# Categorisation of lumbar spine MRI referrals in Denmark as compliant or non-compliant to international imaging guidelines: an inter-rater reliability study

**DOI:** 10.1186/s12998-021-00370-9

**Published:** 2021-03-24

**Authors:** Susanne Brogaard Krogh, Tue Secher Jensen, Nanna Rolving, Malene Laursen, Janus Nikolaj Laust Thomsen, Casper Brink Hansen, Christoffer Høj Werenberg, Erik Rasmussen, Rune Carlson, Rikke Krüger Jensen

**Affiliations:** 1 Department of Diagnostic Imaging, Silkeborg Regional Hospital, Silkeborg, Denmark; 2 Department of Sports Science and Clinical Biomechanics, University of Southern Denmark, Odense, Denmark; 3 Chiropractic Knowledge Hub, Odense, Denmark; 4 DEFACTUM, Central Denmark Region, Aarhus, Denmark; 5 Research Unit, Centre of Elective Surgery, Regional Hospital of Silkeborg, Silkeborg, Denmark; 6 Center for General Practice, Department of Clinical Medicine, Aalborg University, Aalborg, Denmark

**Keywords:** Low back pain, MRI, ACR, Imaging appropriateness criteria, Inter-rater reliability

## Abstract

**Background:**

Managing low back pain (LBP) often involves MRI despite the fact that international guidelines do not recommend routine imaging. To allow us to explore the topic and use this knowledge in further research, a reliable method to review the MRI referrals is needed. Consequently, this study aimed to assess the inter-rater reliability of a method evaluating lumbar spine MRI referrals’ appropriateness.

**Methods:**

Four inexperienced students (chiropractic master’s students) and a senior clinician (chiropractor) were included as independent raters in this inter-rater reliability study. Lumbar spine MRI referrals from primary care on patients (> 18 years) with LBP with or without leg pain were included. The referrals were classified using a modified version of the American College of Radiology (ACR) imaging appropriateness criteria for LBP. Categories of appropriate referrals included; fractures, cancer, previous surgery, candidate for surgery or suspicion of cauda equina. Inappropriate referrals included lacking information on previous non-surgical treatment, no word on non-surgical treatment duration, or “other reasons” for inappropriate referrals.

After two rounds of training and consensus sessions, 50 lumbar spine MRI referrals were reviewed independently by the five raters. Inter-rater reliability was quantified using unweighted Kappa statistics, and the observed agreement was calculated with both a pairwise comparison and an overall five-rater comparison.

**Results:**

Inter-rater reliability was substantial, with a Kappa value for appropriate vs. inappropriate referrals of 0.76 (95% CI: 0.55–0.89). When six and eight subcategories were evaluated, the Kappa values were 0.77 (95% CI: 0.58–0.91) and 0.82 (95% CI: 0.72–0.92), respectively.

The overall percentage of agreement for appropriate and inappropriate referrals was 92% and ranged from 88 to 98% for the pairwise comparisons of the five raters’ results. For the six and eight subcategories, the overall agreement was 92 and 88%, respectively, ranging from 88 to 98% and 84–92%, respectively, for the pairwise comparisons.

**Conclusion:**

The inter-rater reliability of the evaluation of the appropriateness of lumbar spine MRI referrals, according to the modified ACR-appropriateness criteria, was found to range from substantial to almost perfect and can be used for research and quality assurance purposes.

**Supplementary Information:**

The online version contains supplementary material available at 10.1186/s12998-021-00370-9.

## Background

Low back pain (LBP) is the leading cause of disability globally [1]. As many as 80% of all people experience at least one episode of back pain during their lifetime, and LBP is the most common reason for consulting a general practitioner (GP) [2]. LBP accounts for almost 10% of all visits to GPs in Denmark and 30% of all visits to physiotherapy or chiropractic clinics [2, 3]. LBP management’s common practice often includes imaging, even though routine imaging is not recommended by international guidelines [4]. In Denmark, the direct and indirect cost of back pain and back-related disease are estimated to 1,7 billion EUR yearly [2], including imaging costs. A systematic review estimated the mean cost of diagnostic imaging to constitute 7% of the total direct costs of managing LBP [5]. In the past two decades, an overall increase in imaging for LBP has been described in several studies [6–10]. Several factors could influence the increasing number of MRIs, including an increasing elderly population, regional variation (e.g. access to MRI) [11] and practice culture (e.g. attitudes and beliefs of patients and clinicians) [12].

Routine use of MRI for non-specific LBP is considered inappropriate by national and international guidelines [4, 13, 14] as the association between MRI findings and LBP is often weak or inconsistent and does not inform the prognosis or treatment choice [15, 16]. Some suggestions propose that MRI in patients with non-specific LBP can lead to a worse outcome [17, 18]. However, MRI can be a useful tool in managing specific causes of LBP providing the clinician with detailed information of spinal pathology. It is recommended in case of suspicion of “red flags” [19] (i.e. infection, tumour, fracture, or cauda equina syndrome) or when considering surgery [13]. The purpose of imaging guidelines is to inform the GP’s decision as to whether they should refer their patient to MRI. The American College of Radiology (ACR) Imaging Appropriateness Criteria [13] are evidence-based guidelines for LBP developed by a multidisciplinary expert panel. The guideline development and revision includes an extensive analysis of current medical literature from peer-reviewed journals and the application of well-established methodologies [13] to rate the appropriateness of imaging and treatment procedures for specific clinical LBP scenarios.

A referral usually contains factual information including a narrative text which, describes the patient’s condition, e.g., pain localisation and duration, what has been done to help the patient until now, and finally the clinician’s tentative diagnosis. This information helps the radiology department decide whether the patient is eligible for imaging and which modality is most appropriate. A systematic review identified 33 studies investigating imaging referrals’ classification as appropriate or inappropriate, using different methods based on various published guidelines [20]. The results showed an overuse as well as underuse of imaging, suggesting that it is not merely a question of reducing imaging but rather decreasing inappropriate imaging.

To investigate whether MRI referrals from Danish GPs are compliant to guidelines, a reliable method is needed to categorise MRI referrals as appropriate or inappropriate. It is necessary to access the reliability of the method to use it for data collection and for other research project to repeat the method [14].

This study aimed to develop a reliable method for assessing lumbar spine MRI referrals’ appropriateness with respect to international imaging guidelines. The specific objective was to test the inter-rater reliability of extracting data from imaging referrals’ narrative text.

## Methods

This study was reported according to the Guidelines for Reporting Reliability and Agreement Studies (GRRAS) [14].

### Design

Inter-rater reliability study.

### Study population

The study sample consisted of MRI referrals received by a Regional Hospital Silkeborg’s (RHS) radiology department in Denmark in 2016. The referrals concerned patients ≥18 years with LBP with or without leg pain referred for an MRI of the lumbar spine from clinicians in the primary health care sector. In a Danish setting, this includes GPs, consultants (e.g., rheumatology or neurology), and chiropractors in the RHS catchment area. The referrals were received by the radiology department and checked for contraindications for MRI.

During the data collection period, the radiology department’s procedure was to accept referrals from GPs even though the clinical reason for imaging was not appropriate. Some referrals did not contain enough information about absolute MRI contraindications, such as materials not compatible with MRI. If so, the referrals were returned to the clinician to request further information before acceptance. All referrals in this study were of patients who received an MRI of the lumbar spine.

### Data collection

MRI referrals were received and stored in the *Kodak Carestream RIS* (Radiology Information System) version 6.3.0. The narrative texts were exported from the RIS-archive and were de-identified and uploaded to REDCap electronic data capture tools hosted at Aarhus University [21, 22] REDCap (Research Electronic Data Capture).

### Classification of referrals

**Fig. 1:**
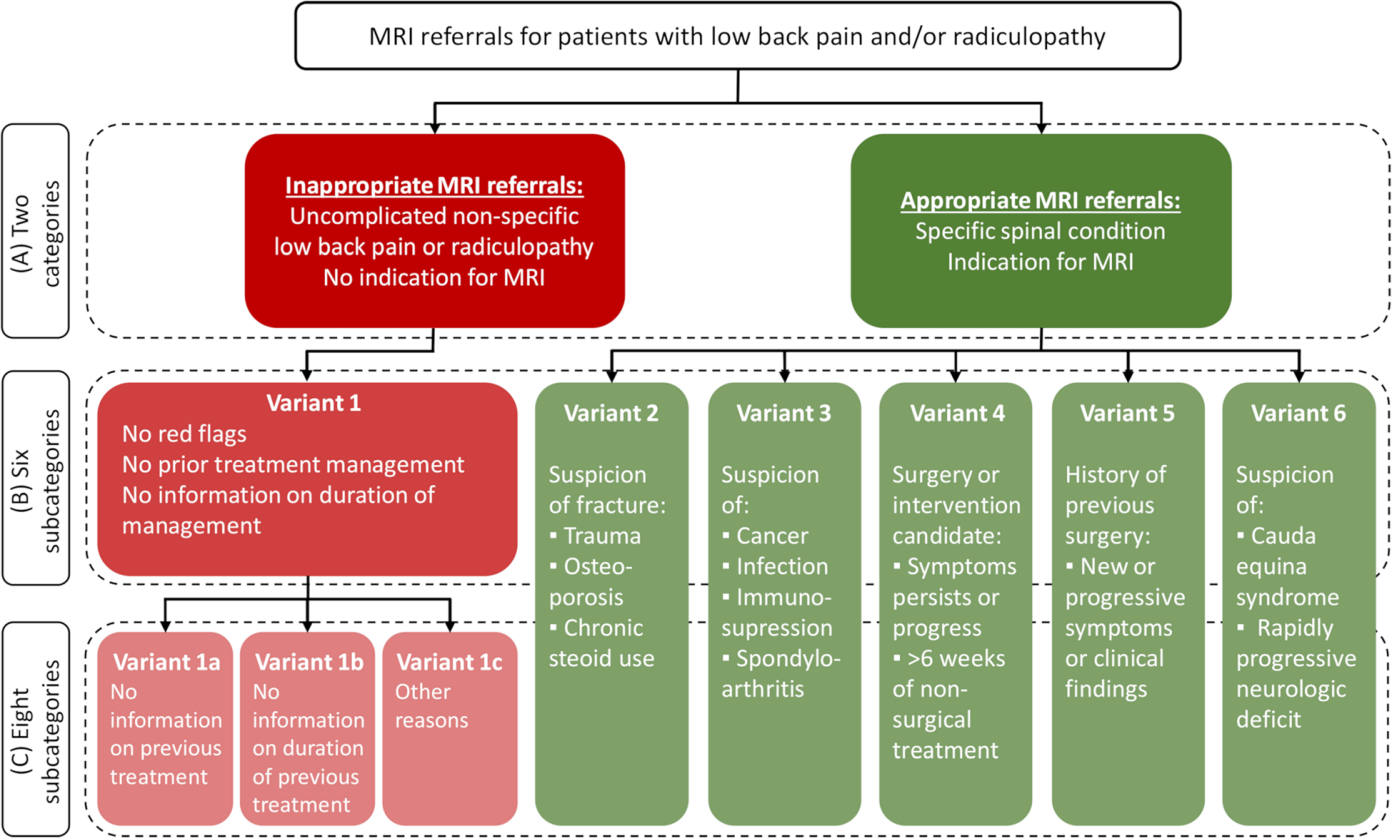
Flow chart for the classification of the referrals according to the modified ACR-appropriateness criteria

Referrals were classified as compliant or non-compliant to the 2015 version of the ACR imaging appropriateness criteria for LBP [13]. The ACR-appropriateness criteria concern MRI referrals for patients with LBP or radiculopathy or both. They describe one scenario of inappropriate MRI referrals (‘Variant 1’) and five scenarios of appropriate MRI referrals (‘Variant 2–6’). A flow chart was created to operationalise the criteria (Fig. 1) further based on these scenarios. If any of the referrals included information on red flags or had a clinical indication of imaging, the referrals were considered appropriate (Fig. 1, green box). These appropriate referrals were subdivided into five categories predefined by the ACR criteria as ‘Variant 2–6’: Variant 2) Suspicion of fracture (e.g. trauma, osteoporosis, chronic steroid use); Variant 3) Suspicion of cancer, infection, immunosuppression or spondylarthritis; Variant 4) Candidate for surgery or intervention with persistent or progressive symptoms during or following six weeks of conservative management; Variant 5) New or progressing symptoms or clinical findings with history of prior lumbar surgery; Variant 6) Suspected cauda equina syndrome or rapidly progressive neurological deficit. If the MRI referrals did not include any of these conditions, the referrals were deemed inappropriate (Fig. 1, red box). For this study’s purpose, the ACR-appropriateness criteria were slightly modified by dividing the inappropriate referrals into three subcategories: 1) no information on previous non-surgical treatment, 2) no information on the duration of non-surgical treatment or 3) other reasons. Details on the classification process are provided in Additional file 1.

Three different classifications of the ACR-appropriateness criteria were evaluated in this study. Firstly, the most important evaluation in a clinical context is whether the MRI referral is appropriate or inappropriate (Fig. 1 (A)). Therefore, the inter-rater reliability of the classification of referrals into these two categories was tested. Secondly, the original ACR-appropriateness criteria were described with six subcategories, of which the five appropriate categories were helpful for the radiology department to decide the most appropriate imaging modality. Therefore, the reliability of these six subcategories was tested (Fig. 1 (B)). Thirdly, as we modified the criteria by dividing the inappropriate referral category into three subgroups (see below), we found it relevant to also test the reliability of this new criteria with eight subcategories in order to inform upcoming research projects (Fig. 1 (C)).

### Raters, training and consensus

Four inexperienced students (chiropractic master students) and a senior clinician (chiropractor) were chosen as independent raters in this inter-rater reliability study. The senior clinician was a part of the research group and had 4 y of experience managing referrals and reading spinal imaging (radiographs and MRI) at the Radiology department at RHS and 15 years of clinical experience with LBP patients in primary and secondary care. The four inexperienced raters were all in their final year (fifth year) of the chiropractic master’s program and had no experience reviewing imaging referrals. Inexperienced raters were chosen to ensure that anyone could perform the rating regardless of clinical knowledge regarding MRI referrals.

Before the inter-rater reliability study, introduction and two training sessions were carried out to ensure consensus regarding the understanding of classification criteria and identify potential practical issues. The ACR-appropriateness criteria were distributed, and a flowchart based on the ACR criteria was presented to the rater group (Fig. 1). For both training sessions, nine and 10 MRI referrals were randomly selected from a sample of approximately 1000 referrals and were independently evaluated by each rater, according to Fig. 1. Each rater’s final classification of the MRI referrals was registered in an Excel worksheet developed for data collection in the present study and based on the categories in Fig. 1.

In the first training session, nine randomly selected referrals were rated, and the raters agreed on the classification of six referrals. During the discussion, it became clear that the disagreement (three referrals) was caused by lacking information from the narrative text from the referrals. In particular, ambiguous or lacking information about non-surgical treatment and non-surgical treatment duration led to subjective assessments by the raters and therefore disagreement. For example, if a referral described a patient who had received physiotherapy ‘several times’ or that the patient had ‘regularly’ performed training, the time duration of non-surgical treatment remained unclear. The raters agreed that the non-surgical treatment modality and the exact timeframe should be explicitly stated to reduce the risk of subjective assessments. As a result of this decision, the three subgroups described in the ‘Methods section’ were added to the ACR-appropriateness model (Fig. 1 (C)). With the modified flow chart, the second training session was conducted with another 10 randomly selected lumbar spine MRI referrals with an agreement of eight out of 10. After discussing the two referrals, the five raters reached consensus on all 10 referrals, and no further training was performed.

### Sample size

The final study sample for the inter-rater reliability study consisted of 50 referrals considered appropriate for this type of study [23]. The referrals were randomly selected from the same sample of 1000 referrals as the training-sessions.

### Data entry and statistical analyses

All five raters independently reviewed and stored data in the data collection sheet. Raters were blinded to the results of their fellow raters. Also, raters were blinded to any other information than tentative diagnosis, date, and the referral’s narrative text.

For all raters, the prevalence of each category was estimated and tabulated. This was done to clarify the potential systematic difference between readers and enable assessment of the sample’s homogeneity based on the tabulation. The comprehensive agreement and expected agreement were calculated with a pairwise comparison of all raters and an overall five-rater comparison. Inter-rater reliability was quantified using Kappa statistics for two raters and Fleiss Kappa statistics based on Cohens Kappa for more than two raters [24]. Kappa values were reported with 95% confidence interval (CI). All calculations were performed for two categories (appropriate versus inappropriate MRI referral classification), six subcategories (one inappropriate and five appropriate MRI referral classifications) and all eight subcategories (three inappropriate and five appropriate MRI referral classifications).

Kappa statistics were interpreted according to the six levels defined by Landis and Koch [25]: < 0.0 ‘Poor’, 0.01–0.20 ‘Slight’, 0.21–0.40 ‘Fair’, 0.41–0.60 ‘Moderate’, 0.61–0.80 ‘Substantial’ and 0.81–1.00 ‘Almost perfect’.

One of the co-authors (RKJ) performed statistical analyses who did not participate in the data collection. Data management and analysis were performed using STATA version 16.0 (StataCorp LLC, TX77845, USA).

## Results

In total, 50 MRI referrals were evaluated by the five raters. The categorisation’s prevalence into appropriate versus inappropriate referrals by each of the raters and the subcategories’ prevalence are displayed in Table 1.

**Table 1:** Prevalence of classification for two, six and eight subcategories for each of the five raters

	Rater 1	Rater 2	Rater 3	Rater 4	Rater 5
**Two categories**:	*n* (%)	*n* (%)	*n* (%)	*n* (%)	*n* (%)
Inappropriate referrals for MRI	41 (82)	40 (80)	39 (78)	40 (80)	42 (84)
Appropriate referrals for MRI	9 (18)	10 (20)	11 (22)	10 (20)	8 (16)
**Six subcategories**	*n* (%)	*n* (%)	*n* (%)	*n* (%)	*n* (%)
*Inappropriate referrals for MRI*					
1 No information on previous treatment and duration	41 (82)	40 (80)	39 (78)	40 (80)	42 (84)
*Appropriate referrals for MRI*					
2 Fracture	0 (0)	0 (0)	0 (0)	0 (0)	0 (0)
3 Cancer, infection, immunosuppression or spondylarthritis	5 (10)	5 (10)	6 (12)	5 (10)	4 (8)
4 Persistent/progressive symptoms after six weeks of treatment	0 (0)	2 (4)	1 (2)	1 (2)	0 (0)
5 Prior lumbar surgery and new or progressing symptoms	4 (8)	3 (6)	4 (8)	4 (8)	4 (8)
6 Cauda equina syndrome / progressive neurologic deficit	0 (0)	0 (0)	0 (0)	0 (0)	0 (0)
**Eight subcategories:**	*n* (%)	*n* (%)	*n* (%)	*n* (%)	*n* (%)
*Inappropriate referrals for MRI*					
1 No information on previous treatment	23 (46)	24 (48)	20 (40)	25 (50)	26 (52)
2 No information on duration of previous treatment	18 (36)	16 (32)	19 (38)	15 (30)	16 (32)
3 Other reasons	0 (0)	0 (0)	0 (0)	0 (0)	0 (0)
*Appropriate referrals for MRI*					
4 Fracture	0 (0)	0 (0)	0 (0)	0 (0)	0 (0)
5 Cancer, infection, immunosuppression or spondylarthritis	5 (10)	5 (10)	6 (12)	5 (10)	4 (8)
6 Persistent/progressive symptoms after six weeks of treatment	0 (0)	2 (4)	1 (2)	1 (2)	0 (0)
7 Prior lumbar surgery and new or progressing symptoms	4 (8)	3 (6)	4 (8)	4 (8)	4 (8)
8 Cauda equina syndrome / progressive neurologic deficit	0 (0)	0 (0)	0 (0)	0 (0)	0 (0)

The overall observed agreement for the two category MRI referral classification (appropriate versus inappropriate) was 92.4% for the five-rater comparison and ranged from 88 to 98% for the pairwise comparison (Table 2). The interrater reliability was ‘Substantial’ with a Kappa value of 0.76 (95% CI: 0.55–0.89). The pairwise rater comparison ranged from 0.63 (95% CI: 0.36–0.90) to 0.94 (95% CI: 0.82–1.00) (Table 2). When the six categories from the original ACR-appropriateness criteria was accessed, the observed agreement for the five-rater comparison was 92.4% and ranged from 88 to 98% (Table 3). The Kappa value for the five-rater comparison was 0.77 (95% CI: 0.58–0.91) (‘Substantial’) and the pairwise rater comparison ranged from 0.65 (95% CI: 0.36–0.90) to 0.94 (95% CI: 0.77–1.00) (Table 3). For the eight subcategories, the overall five-rater observed agreement was 88% and ranged from 84 to 92% for the pairwise comparison (Table 4). The five-rater comparison was ‘Almost perfect’ with a Kappa value of 0.82 (95% CI: 0.72–0.91), and the pairwise comparison ranged from 0.76 (95% CI: 0.59–0.90) to 0.87 (95% CI: 0.73–0.97) (Table 4).

**Table 2:** Interrater reliability for categorisation of appropriate and inappropriate referrals for MRI (*n* = 50)

Raters	Observed agreement (%)	Expected agreement (%)	Kappa (95% confidence interval)
1:2	90.0	67.5	0.68 (0.41–0.94)
1:3	88.0	67.9	0.63 (0.36–0.90)
1:4	90.0	69.2	0.68 (0.41–0.94)
1:5	94.0	71.8	0.79 (0.56–1.00)
2:3	90.0	66.8	0.70 (0.45–0.95)
2:4	92.0	68.0	0.75 (0.52–0.98)
2:5	92.0	70.4	0.73 (0.48–0.98)
3:4	98.0	66.8	0.94 (0.82–1.00)
3:5	94.0	69.0	0.81 (0.60–1.00)
4:5	96.0	70.4	0.87 (0.68–1.00)
1:2:3:4:5	92.4	69.0	0.76 (0.55–0.89)

**Table 3:** Inter-rater reliability for categorisation of appropriate and inappropriate referrals for MRI with six subcategories (*n* = 50)

Raters	Observed agreement (%)	Expected agreement (%)	Kappa (95% confidence interval)
1:2	90.0	67.1	0.70 (0.40–0.92)
1:3	88.0	65.8	0.65 (0.36–0.90)
1:4	90.0	67.2	0.70 (0.40–0.92)
1:5	94.0	70.3	0.80 (0.46–1.00)
2:3	90.0	64.2	0.72 (0.45–0.94)
2:4	92.0	65.6	0.77 (0.44–0.94)
2:5	92.0	68.5	0.75 (0.47–0.94)
3:4	98.0	64.3	0.94 (0.77–1.00)
3:5	94.0	67.1	0.82 (0.56–1.00)
4:5	96.0	68.6	0.87 (0.65–1.00)
1:2:3:4:5	92.4	66.9	0.77 (0.58–0.91)

**Table 4:** Inter-rater reliability for categorisation of appropriate and inappropriate referrals for MRI with eight subcategories (*n* = 50)

Raters	Observed agreement (%)	Expected agreement (%)	Kappa (95% confidence interval)
1:2	88.0	35.1	0.82 (0.65–0.94)
1:3	84.0	33.9	0.76 (0.59–0.90)
1:4	88.0	35.4	0.81 (0.65–0.93)
1:5	90.0	36.9	0.84 (0.70–0.97)
2:3	84.0	33.1	0.76 (0.61–0.90)
2:4	88.0	35.2	0.82 (0.68–0.94)
2:5	92.0	36.5	0.87 (0.73–0.97)
3:4	90.0	33.2	0.85 (0.71–0.97)
3:5	86.0	34.6	0.79 (0.63–0.92)
4:5	92.0	37.0	0.87 (0.70–0.97)
1:2:3:4:5	88.2	35.1	0.82 (0.72–0.92)

## Discussion

This study is the first reliability study of appropriate and inappropriate lumbar spine MRI referrals compliant to international imaging guidelines to the best of our knowledge. The overall inter-rater reliability for any of the three types of classification ranged from ‘Substantial’ to ‘Almost perfect’.These findings suggest that the appropriateness of MRI referrals for LBP can be categorised satisfactorily.

Two studies have previously used inexperienced raters’ technique extracting data from narrative MRI diagnostic radiology reports, on lumbar and cervical MRI [26, 27]. Both studies found ‘Substantial’ to ‘Almost perfect’ agreement depending on the specific MRI variable (e.g., disc herniation, foraminal stenosis, degeneration). These findings are in accordance with our results and support the assumption that unstructured text data (narrative reports) can be quantified in a low tech and cheap way. Detecting the presence of a word in an unstructured text is also technically possible using electronic ‘natural language processing’. Pons et al. [28] conducted a systematic review in 2016 and found 67 publications describing the extraction of information from unstructured texts and concluded that the performance was generally high. Natural language processing is based on predefined words or phrases, which is also the present study’s methodological framework, which found a comparable high performance when extracting information from unstructured texts.

A systematic review from 2019 [29] clarifies the complexity of the wide range of information from the electronic health records. The study concludes that more focus is needed on methods for extracting symptom information from electronic health records and using the symptom information for disease classification rather than examining symptoms themselves. The ACR criteria used in the present study focus on both disease classification (e.g., fracture, cancer, infection, spondylarthritis) but also on symptoms and management (e.g., progressive neurologic deficit, persistent/progressive symptoms after six weeks of treatment). During the training session, it became clear that the extraction of information about symptoms and management was less intuitive than disease classification, which is in line with the review’s conclusions [29].

### Methodological considerations

The 95% CI of the overall inter-rater reliability for appropriate and inappropriate MRI referrals ranged from 0.55 (Moderate) to 0.89 (Almost perfect) including two levels on the Landish and Koch scale, which could imply that a larger sample would have been suitable. Instead of running a sample size calculation before conducting the study, the sample was based on recommendations for reliability studies [23] which might be considered a limitation. However, an overall agreement of ‘Moderate’ (lower CI) would still be regarded as sufficient to support future data collection using this method.

The heterogeneity of data from a narrative text makes the transformation into quantitative data challenging. As we chose to use inexperienced raters (students) in this study, the results may not apply to a clinical setting with more experienced raters. However, this was a deliberate decision to increase the likelihood that the wording of the narrative text matched the criteria rather than an interpretation made by the assessor originating from clinical experience. Also, we included one experienced rater in the team to teach the students and ensure a high scientific standard. Looking at the prevalence estimates, there was no systematic difference between the experienced rater (Rater 1) and the students (Raters 2–5), see Table 1. Yet, it was inevitable that some degree of interpretation of the text by the rater would occur. However, the interpretation of narrative text reflects the everyday clinical practice in most radiology departments.

There are two crucial assumptions to consider when classifying lumbar spinal MRI referrals into appropriate or inappropriate referrals. First, we presume that the clinicians who have seen and clinically evaluated the patient have done so according to the highest clinical standard. They have written a narrative imaging referral in the scope of current guidelines. Second, the clinician who reviews the spine MRI referrals, and who has never seen or examined the patient must extract the required information from the referral to assess whether it is an appropriate or inappropriate referral according to guidelines. Further research with a qualitative approach is necessary to obtain a deeper understanding of the complexity of the steps in this process.

Future studies need to investigate the present compliance to guidelines and barriers and facilitators for clinicians to be guideline compliant to ensure that MRI referrals are guideline compliant and possible influence future use of imaging. The results of this study suggest a low tech, cheap and reliable data collection method of narrative text imaging referrals for MRI.

## Conclusion

According to ACR appropriateness criteria, the inter-rater reliability of categorising inappropriate and appropriate lumbar spine MRI referrals was found to be substantial to almost perfect. This method may, therefore, be used for research and quality assurance purposes in future research.

## Supplementary Information

**Additional file 1:.** Categorisation of the imaging referrals

## Data Availability

The dataset used in the current study is available from the corresponding author on reasonable request.

## Abbreviations

ACR: American College of Radiology
CI: Confidence interval
LBP: Low back pain
MRI: Magnetic resonance imaging
