# Supplementary material for: Categorisation of lumbar spine MRI referrals in Denmark as compliant or non-compliant to international imaging guidelines: an inter-rater reliability study
Source: Chiropr Man Therap. 2021 Mar 24;29:12. doi: 10.1186/s12998-021-00370-9 (PMC7988995; doi:10.1186/s12998-021-00370-9)
Supplement: Supplementary file 1 — Additional file 1:. Categorisation of the imaging referrals [file 12998_2021_370_MOESM1_ESM.docx]

**Additional file 1: Categorisation of the imaging referrals**

This method for categorising imaging referrals was designed to to ensure concencus between clinicians or trainees regardless of clinical knowledge regarding MRI referrals. After one introduction meeting and two training session the was tested for inter-rater reliability and the overall percentage of agreement for appropriate and inappropriate referrals was 92%.

We used five raters: four inexperienced and one experienced rater. The flow chart below is a modified version of the ACR-criteria and is used to categorise the imaging referrals.


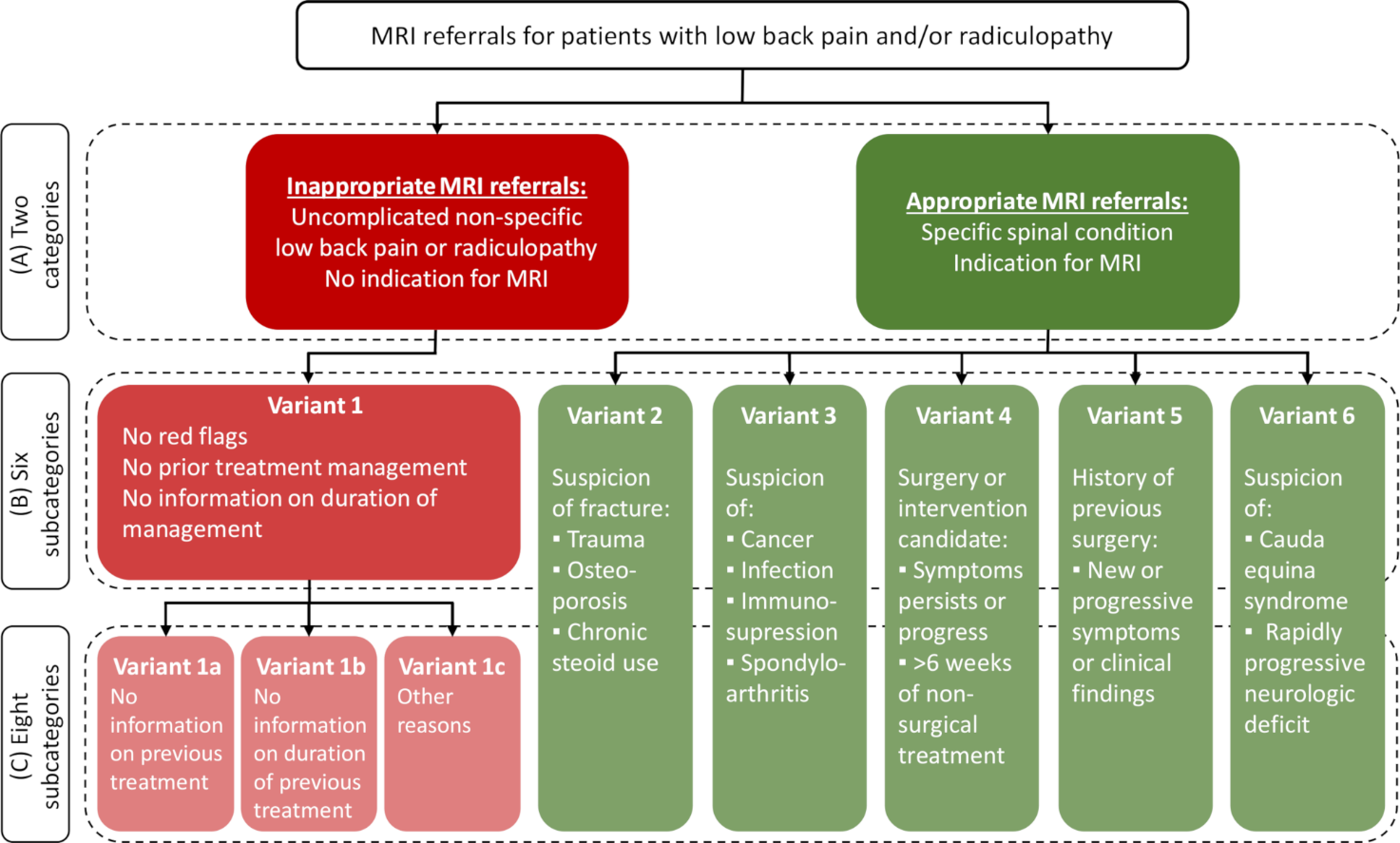


The narrative text from the referrals will inform the flow chart and divide the referrals into guideline compliant “appropriate” (green, variant 2-6) or not guideline compliant “inappropriate” (red, variant 1a, 1b and 1c) referrals.

**Introductory meeting.**

An introductory meeting was held by the most experienced rater to introduce the ACR-criteria and to discuss how to use the flow chart.

At the meeting, the raters agreed to expand variant 3 with suspicion of spondylarthritis (SpA), because MRI is the first choice in the diagnosis of SpA. Furthermore, the raters discussed variant 4. The raters specified that variant 4 must describe persistent symptoms after six weeks of non-surgical treatment (training, manual treatment, etc.). Furthermore, the referral must describe the need for further evaluation, such as surgical or an expanded medical examination (read ACR-criteria, variant 4: intervention candidate). In a Danish setting that would be a medical evaluation at a public regional spine centre. If the non-surgical treatment period is unclear or if no further assessment seems to be needed, the referral should instead be categorised as inappropriate (variant 1).

**Training session one**

Nine referrals were distributed among the raters, and the raters independently categorised the referrals in an excel sheet. The experienced rater compared the result and found the main disagreement between variant 1 and 4. The differences between raters were found concerning weak descriptions related to the time frame. E.g. if the referral read, “the patient has received physio treatment for a long time” or “the patient has been persistent with her training”. In those cases, some degree of interpretation occurred among the raters.

To accommodate and avoid disagreement the raters added three subvariants: 1a (non-surgical treatment was not described), 1b (no time frame of non-surgical treatment) and 1c (referrals that did not meet the inclusions criteria). These subvariants made it possible for the raters to categorise referrals that lacked information about the non-surgical treatment, and raters agreed not to interpret or ‘read between the lines’ when the information was missing.

**Training session two**

To ensure agreement on the new definitions was reached, a second training round was held. Ten referrals were distributed among the raters, and the raters independently categorised the referrals in an excel sheet. The experienced rater compared the result and found a few individual disagreements, based on overlooked information from the referrals. An agreement was reached by discussion in the group, and no more training sessions were held. More training sessions should be considered if raters do not reach an agreement.

**Considerations concerning the appropriate guideline variants:**

Variant 2:

If the referral describes any of the following clinical symptoms:

- Spinal fracture, any circumstances that could entail fracture.
- Patients with osteopenia or osteoporosis.
- Any long-term steroid use.

Variant 3:

If the referral describes any of the following clinical symptoms:

- Known cancer disease or any suspicion of metastases to the spine.
- Suspicion of spinal infection (discitis or osteomyelitis).
- Known immunosuppression condition.
- Diagnosed with or clinical suspicion of spondylarthritis (SpA).

Variant 4:

If the referral describes any of the following clinical symptoms:

- Persistent symptoms after six weeks of non-surgical treatment (pain medication, training, manual treatment etc.) and therefore needed further evaluation at a surgeon or an extended medical examination (DK: public spine centre or medical specialist).
- If the non-surgical treatment is not described with an exact time frame (at least six weeks) the raters categorised the referral in-appropriate.

Variant 5:

If the referral describes any of the following clinical symptoms:

- Previously had surgery combined with new or progressive symptoms (pain and disability were enough, not necessarily neurological symptoms) in the same region as the former surgery.

Variant 6:

If the referral describes any of the following clinical symptoms:

- Progressive neurological symptoms such as “the patient developed drop foot from last consultation” or cauda equina symptoms (urine retention or sphincter symptoms).

**Considerations concerning the guideline in-appropriate variants:**

If the referral did not contain any information about the conditions in variant 2-6, the referral was categorised in-appropriate, variant 1.

With the three subvariants (1a, 1b and 1c), the raters are given the possibility to categorise the specific kind of missing information.

Variant 1a:

If the referral had missing information about:

- non-surgical treatment. Some referrals describe long term or recurrent spinal pain, and it could be tempting to assume that some non-surgical treatment has been provided. However, if treatment is not mentioned in the referral, it cannot be categorised as appropriate and will be categorised as inappropriate / 1a.

Variant 1b:

If the referral had missing information about:

- the time frame of non-surgical treatment. Some referrals describe what kind and non-surgical treatment the patient has received but nothing about the time frame. E.g. “the patient has received physiotherapy several times” or that the patient had performed “regularly” training. If treatment is mentioned but not the time frame the referral is categorised as variant 1b.

Variant 1c:

- Referrals that did not meet the inclusions criteria, e.g. spinal pain from another region than the lower back, organic pain or patients under the age of 18 years.
